# Trial of food allergy IgE tests for Eczema Relief (TIGER): study protocol

**DOI:** 10.1093/skinhd/vzag085

**Published:** 2026-07-03

**Authors:** Dominika Corejova, Caroline Pope, Katherine Joyce, Anna Gilbertson, Stephanie MacNeill, Yumeng Liu, Rosie Harris, Kirsty Garfield, Jo Coast, Ingrid Muller, Xiao-Yang Hu, Sophia Williams, Gabriele Vojt, Rosan Meyer, Isabel Skypala, Hannah Morgans, Shoba Dawson, Hannah Wardman, Thomas Blakeman, Sara Brown, Robert Boyle, Hywel Williams, Miriam Santer, Matthew J Ridd

**Affiliations:** Bristol Trials Centre, University of Bristol, Bristol, UK; Bristol Trials Centre, University of Bristol, Bristol, UK; Exeter Clinical Trials Unit, University of Exeter, Exeter, UK; Centre for Applied Excellence in Skin & Allergy Research, University of Bristol, Bristol, UK; Bristol Trials Centre, University of Bristol, Bristol, UK; Bristol Trials Centre, University of Bristol, Bristol, UK; Bristol Trials Centre, University of Bristol, Bristol, UK; Bristol Trials Centre, University of Bristol, Bristol, UK; Health Economics & Health Policy at Bristol, University of Bristol, Bristol, UK; Health Economics & Health Policy at Bristol, University of Bristol, Bristol, UK; Primary Care Research Centre, University of Southampton, Southampton,UK; Primary Care Research Centre, University of Southampton, Southampton,UK; Primary Care Research Centre, University of Southampton, Southampton,UK; Primary Care Research Centre, University of Southampton, Southampton,UK; Department of Medicine, KU Leuven, Leuven,Belgium; Royal Brompton & Harefield NHS Foundation Trust, Part of Guy’s & St Thomas’ NHS Foundation Trust, London, UK; Department of Inflammation and Repair, Imperial College, London, UK; Centre for Applied Excellence in Skin & Allergy Research, University of Bristol, Bristol, UK; Bristol Trials Centre, University of Bristol, Bristol, UK; Division of Immunology, Immunity to Infection and Respiratory Medicine, University of Manchester, Manchester University NHS Foundation Trust, Manchester, UK; Centre for Primary Care & Health Services Research, University of Manchester, Manchester, UK; Institute of Genetics and Cancer, University of Edinburgh, Edinburgh, UK; Mohn Centre for Children’s Health and Wellbeing, School of Public Health, Imperial College, London, UK; Centre of Evidence-Based Dermatology, Lifespan and Population Health, University of Nottingham, Nottingham,UK; Primary Care Research Centre, University of Southampton, Southampton,UK; Centre for Applied Excellence in Skin & Allergy Research, University of Bristol, Bristol, UK

## Abstract

**Background:**

Food allergy may contribute to eczema, but there is variability in the use of tests to investigate this, and uncertainty about possible benefits or harms. The objective is to determine the clinical and cost-effectiveness of test-guided dietary advice compared with standard care for eczema management.

**Methods:**

This is a pragmatic, multicentre, parallel group, individually randomized controlled trial with internal pilot and nested genetic substudy and economic and process evaluations. Children (aged 3 months to <2 years) were recruited via general practitioner surgeries in England and randomized 1:1 to standard care or intervention. Both groups received a ‘Good eczema care’ leaflet. The intervention group also received dietary advice for cow’s milk, hen’s eggs, wheat and soya, based on skin prick tests and dietary history.

**Discussion:**

The primary outcome is parent-reported eczema control [RECap for AtoPic eczema (RECAP)], completed 4-weekly over 24 weeks. Secondary outcomes include eczema symptoms; quality of life; adverse events; breastfeeding status and diet; growth; and parental anxiety. The primary analysis is a multilevel mixed model framework with observations over time nested within participants. A cost–utility analysis will compare quality-adjusted life years for the child and main carer to costs incurred by the National Health Service (NHS) between groups. Additionally, costs from NHS and non-NHS perspectives will be related to a range of outcomes in a cost–consequences approach. A nested process evaluation will assess intervention fidelity, clarify causal mechanisms and identify contextual factors associated with variation in outcomes. This trial will fill an evidence gap of clinical importance and reduce variation in practice and associated harms.

**Protocol registration:**

The protocol is registred with ISRCTN (ISRCTN52892540): https://www.isrctn.com/ISRCTN52892540.

## Teaser

This study protocol describes a primary care trial designed to answer the research question, ‘Does food allergy test-guided dietary advice improve disease control in children with eczema?’

Eczema (also known as atopic eczema or dermatitis) is a common, long-term condition that affects ∼20% of children.^1^ It is characterized by dry, itchy skin and has a strong familial (genetic) basis.^2^

Effective management includes the daily use of topical treatments, but treatment adherence can be challenging.^3^ One reason for this is that parents or carers (hereafter, parents) think that they are treating the symptoms and not the cause.^4^ Parents may worry that an undiagnosed food allergy is the cause for their child’s eczema symptoms, often based on information they have found online.^5^

Between 40% and 75% of parents report excluding food from their child’s diet to try to reduce the symptoms of eczema.^6,7^ Restrictions are commonly implemented without discussing them with a health professional.^8^ Delaying the introduction of or excluding food may contribute to nutritional deficiencies, reduced dietary diversity,^9^ feeding difficulties^10,11^ and/or food allergy development.^12^ Parents who do discuss the role of diet in eczema with their doctor often ask about allergy testing to guide dietary exclusions.^13^

Food allergy is more common in children with eczema.^14^ Children with earlier onset, persistent and severe disease are at increased risk of immediate (IgE-mediated) food allergies,^15^ causing symptoms within 2 h of ingestion. However, most parents are concerned about possible delayed (non-IgE-mediated) reactions, causing their child’s eczema to worsen hours-to-days after ingestion. The relapsing–remitting nature of eczema makes causal associations difficult to identify, especially for foods that are ubiquitous.

There are no specific diagnostic tests for delayed food allergies and, in theory, the use of IgE-specific blood or skin prick tests (SPTs) to investigate non-IgE-mediated reactions is a nonsequitur. Oykhman *et al*.^16^ recently undertook a systematic review of studies comparing dietary elimination vs. no dietary elimination for the treatment of eczema. They identified 10 randomized controlled trials, 4 of which excluded foods based on food allergy tests and/or oral food challenge (OFC). There was low-certainty evidence that dietary elimination slightly improved eczema severity, pruritus and sleep disturbance. However, there was no evidence of any differences based on elimination strategy (empirical vs. guided by testing) or food-specific sensitization. There were insufficient data on any associated harms.

It is therefore unclear whether test-guided dietary decisions improve eczema symptoms or negatively affect children by unnecessarily reducing dietary choices^14^ and distract from the use of conventional treatments. Research is needed to determine whether test-guided dietary modifications improve eczema symptoms.

The aim of this study was to determine the clinical and cost effectiveness of test-guided dietary advice vs. standard care, for the management of eczema. The objectives were as follows: to evaluate the clinical and cost effectiveness of test-guided dietary advice in children with eczema; to assess adherence to and the safety of test-guided dietary advice in children with eczema; and to identify subgroups (through family history or with genetic risk variants) who may preferentially benefit from food allergy testing.

## Patients and methods

The current protocol is version 9.0 (8 December 2025). Practice and participant recruitment are summarized in Figure 1.

**Figure 1:**
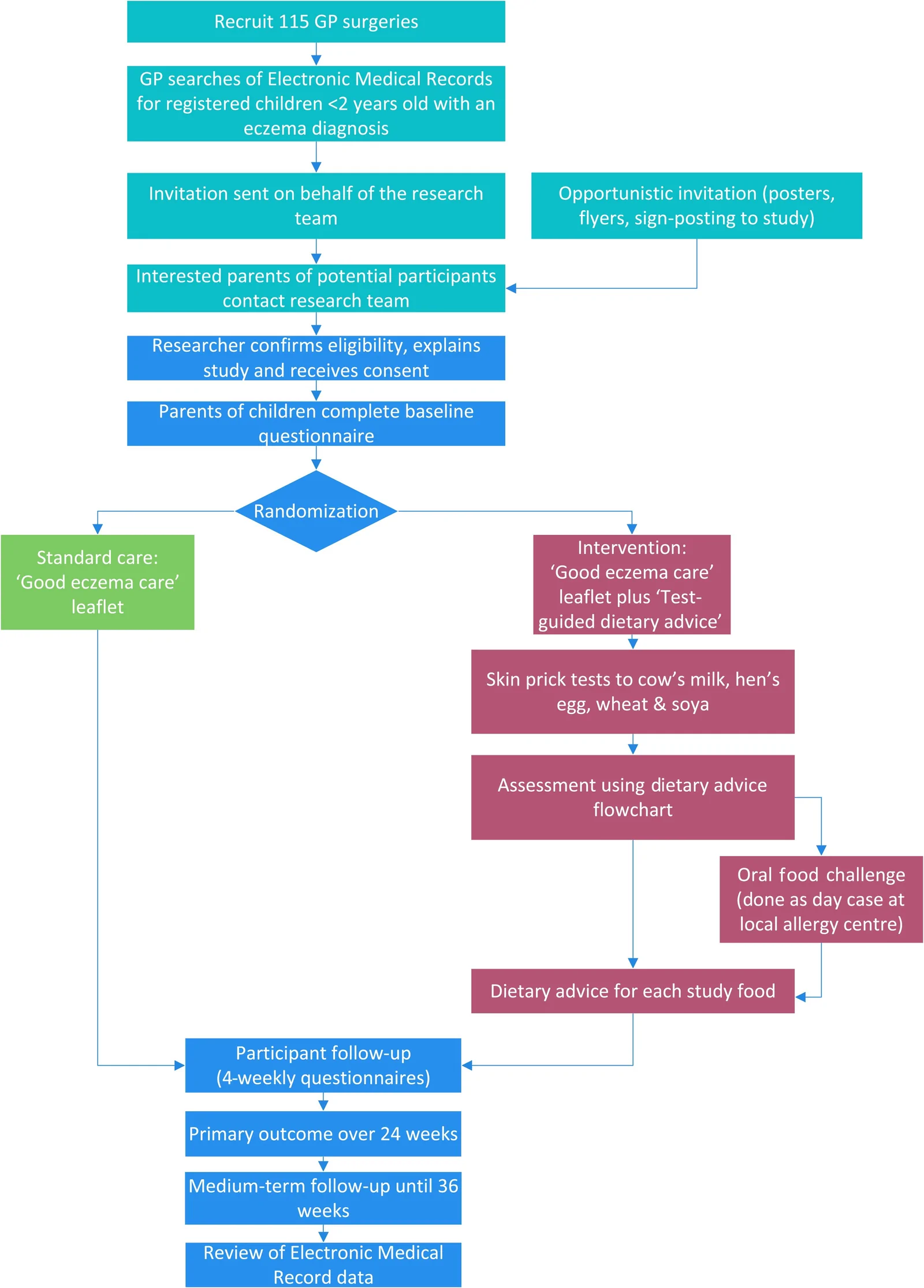
Flow of participants through study. GP, general practitioner.

### Trial design

TIGER (Trial of food allergy IgE tests for Eczema Relief) is a pragmatic, multicentre, parallel group, individually randomized, controlled superiority trial, with internal pilot phase, nested health economic and process evaluations. The internal pilot ran from 1 March 2023 to 31 October 2023, and participant recruitment ended on 22 August 2025.

### Study setting

Participants were recruited from general practitioner (GP) surgeries in four National Institute for Health Research (NIHR) Clinical Research Networks (CRNs) (Greater Manchester, Thames Valley and South Midlands, West of England, Wessex) in England. GP sites also provided a consultation room and medical cover for baseline visits.

Children needing OFCs received them as a day case at a participating allergy centre in Bristol, Manchester, Oxford or Southampton.

### Eligibility criteria

Participant inclusion and exclusion criteria are listed in Table 1.

**Table 1:** Participant inclusion and exclusion criteria

To be eligible, children must:
be aged between 3 months and less than 2 years; and
have eczema diagnosed by an appropriately qualified healthcare professional; and
have mild, moderate or severe eczema (POEM score >2 within the previous 28 days); and
be accompanied by an adult who can give consent
Ineligible children have:
confirmed or probable^a^ immediate (IgE-type) food allergy to the study foods; and/or
previous SPT or IgE blood test for the study foods; and/or
another child in the household already taking part in the trial

POEM, Patient Orientated Eczema Measure; SPT, skin prick test. ^a^Parents who report symptoms, which in the opinion of the allergy panel/their general practitioner, are suspicious of an immediate-type reaction.

### Participant recruitment

Participants were identified through two routes:

- Electronic medical records (EMR) searches for children 3 months to <2 years of age with history of eczema. Children were screened, with reasons for exclusion documented. Parents of potentially eligible families were sent postal invitations and a text or telephone call reminder 2 weeks later.
- Opportunistic: some parents contacted the research team after seeing advertisements at their GP surgery or after discussion with their clinician. GP surgeries were offered computer ‘pop-ups’ that appeared when a relevant diagnostic code was entered.

Interested parents completed a questionnaire, which included the Patient-Orientated Eczema Measure (POEM). For children who were ineligible due to POEM score ≤2, permission was sought to follow them up in case symptoms worsened.

### Data collection

Eligibility checks, consent and prebaseline appointments were delivered by the researcher over the telephone to the parent. Baseline visits took place in the GP surgery. Follow-up appointments were scheduled according to parental preference, usually in the family home.

Researchers administered the prebaseline questionnaire to all participants, and post-home dietary trial of inclusion/exclusion (4–6 weeks after) and OFC (1 week after) questionnaires to ­intervention participants, when applicable. Dietary history of possible immediate and/or delayed symptoms when the child last consumed each of the trial foods (cow’s milk, hen’s egg, wheat, soya) were collected prebaseline and checked before randomization. Ambiguous cases were discussed with an allergy panel before proceeding to randomization. Parents self-completed questionnaires at prebaseline, baseline and every 4 weeks thereafter for 36 weeks (Table 2), online (with email and text reminders) or on paper (with text reminders). For missing questionnaires, an option to complete these over the telephone was offered.

**Table 2:** Schedule of data collection

	Topic	Measure	Week											
			B–	B	B + 4	B + 8	B + 12	B + 16	B + 20	B + 24	B + 28	B + 32	B + 36	B > 36
1	Expression of interest		●											
2	Characteristics of child and family^a^	Bespoke	○											
3	Diet^b^	Infant Feeding Survey/bespoke	○	○	●	●	●	●	●	●	●	●	●	
4	Child quality of life (disease specific)	IDQOL	●							●			●	
5	Child quality of life (generic)	CHU-9D	●				●			●			●	
6	Parent quality of life	EQ-5D-5L and EQ-VAS	●				●			●			●	
7	Parent quality of life	CarerQol	●							●			●	
8	Parental anxiety	GAD-7	●							●			●	
9	Eczema control	RECAP		●	●	●	●	●	●	●	●	●	●	
10	Eczema symptoms	POEM		●	●	●	●	●	●	●	●	●	●	
11	Itch severity	PP-NRS		●	●	●	●	●	●	●	●	●	●	
12	Parent-reported global eczema severity	Global eczema severity		●	●	●	●	●	●	●	●	●	●	
13	Use of topical treatments for eczema	Bespoke		●	●	●	●	●	●	●	●	●	●	
14	Eczema/food allergy genes	DNA saliva sample		▾						▾				
15	Growth of child	Weight, height and head circumference		○						○				
16	Eczema signs	EASI		○						○				
17	Food sensitization	Skin prick tests		◻										
18	Food allergy	(Oral food challenge)		◊										
19	Home dietary trial outcome				◻									
20	Health service utilization	ModRUM					●			●			●	
21	Out-of-pocket expenses/time off work	Bespoke					●			●			●	
22	Trial participation experience	(Exit questionnaire)											●	
23	GP consultations, prescriptions and referrals	EMR notes review												●

B–, prebaseline visit; B, baseline visit; open circles, researcher administered; filled circles, self-complete questionnaire; triangle, optional, collected at either baseline or 24-week appointments; squares, participants in intervention group only; diamonds, participants in intervention group only with equivocal/abnormal allergy results. CarerQol, Care Related Quality of Life; CHU-9D, Child Health Utility 9D scale; EASI, Eczema Area Severity Index; EQ-5D-5L, EuroQol-5 Dimension; EQ-VAS, EuroQol Visual Analogue Scale; GAD-7, Generalised Anxiety Disorder 7; GP, general practitioner; IDQOL, Infant Dermatitis Quality of Life; ModRUM, modular resource-use measure; POEM, Patient-Oriented Eczema Measure; PP-NRS, Peak Pruritis Numerical Rating Scale; RECAP, RECap for AtoPic eczema. ^a^Participant demographics and medical history, UK eczema diagnostic criteria for atopic dermatitis, family history of atopy, parent health literacy [Single Item Literary Screening (SILS)] and prior expectations. ^b^Diet of child and breastfeeding mother, if applicable.

Objective assessments of the participant’s skin [Eczema Area Severity Index (EASI)] and anthropometric data (weight, length and head circumference) were collected at baseline and 24 weeks (±10 days). An optional saliva sample for DNA analysis was collected by the researcher at the baseline or 24-week appointment.

At the end of the study, participants’ primary care EMRs were reviewed (from birth until 36 weeks after the date of randomization) and data were extracted on prescriptions, consultations and referrals.

### Allocation

Subject to participants being well, not having had an oral anti­histamine in the previous 72 h, and having clear skin on the ­forearm or back for skin prick testing, participants were allo­cated at the baseline visit. An online randomization system (www.sealedenvelope.com) assigned children to standard care or test-guided dietary advice groups (1:1 ratio), stratified by eczema severity (POEM) and centre (Bristol, Southampton or Manchester). Nobody doing this had access to the random allocation sequence.

### Standard care

All participants were given a ‘Good eczema care’ leaflet (see Supporting Information), designed to standardize, but not enhance, care across groups. It was not supplemented with any additional advice.

Participants’ GPs retained clinical responsibility and care was otherwise unchanged. This means clinicians were free to change treatments, undertake investigations and/or make referrals (including for possible food allergy).

### Intervention

Participants in the intervention group received test-guided dietary advice about cow’s milk, hen’s egg, wheat and soya, based on SPTs and dietary history. This was delivered by trained researchers who administered and interpreted the SPTs.

- Lancets were used to prick drops of reagents and positive and negative controls placed on the skin, into the epidermis and superficial dermis (Table 3). The diameter of any wheal reaction was measured in millimetres after 15 min.
- A flowchart (Figure 2) was used to determine the dietary advice and provisionally inform the parent of the outcome for each food at the appointment. This advice was ratified by a clinician on the research team before being confirmed in writing to both the parent and their GP.

One of the following recommendations was provided for each food.

**Figure 2:**
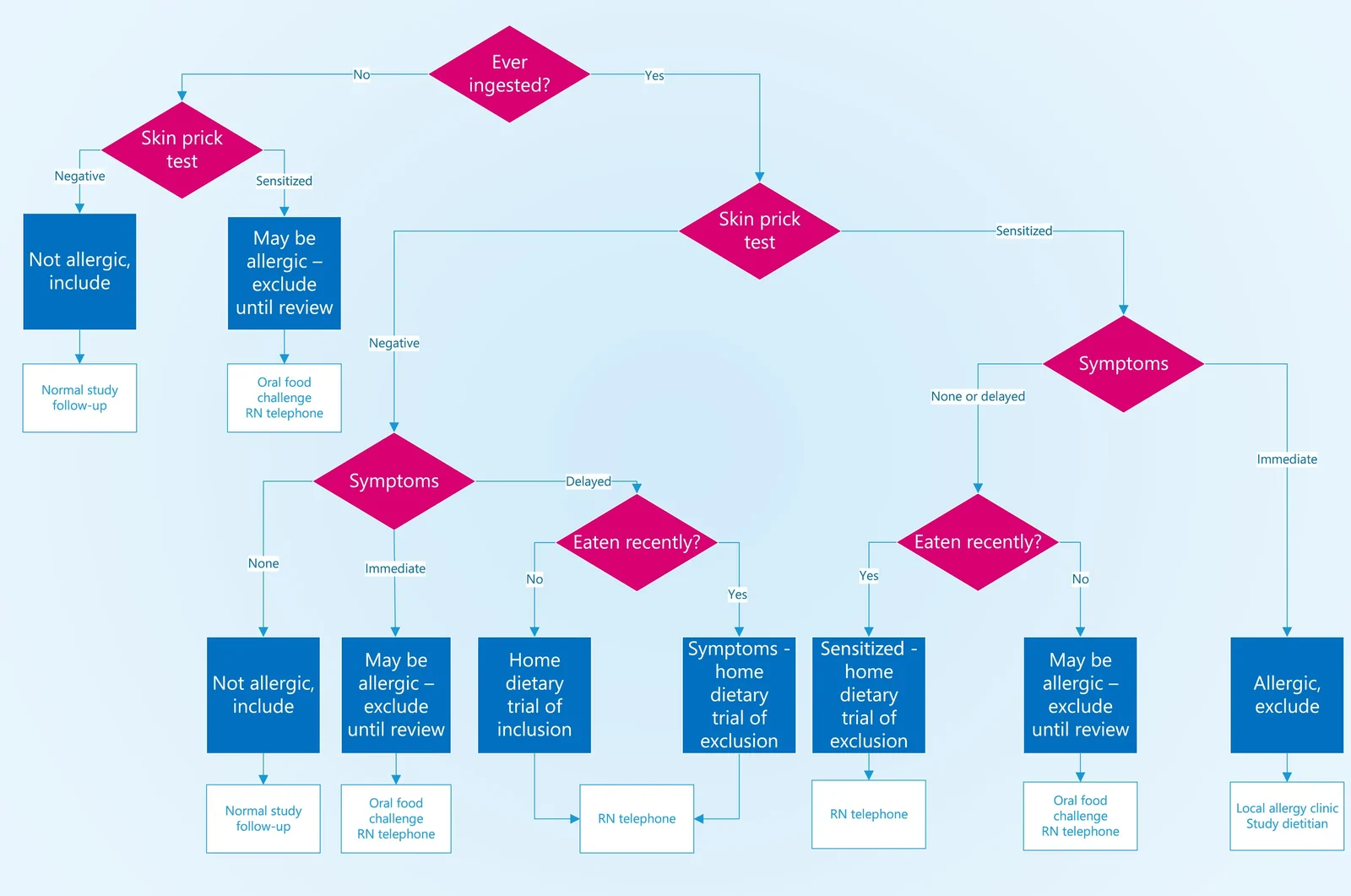
Dietary advice flowchart. Immediate symptoms are those (worsening of eczema; hives; itching; swelling around the face, lips or eyes; abdominal pain; vomiting; swelling of tongue or airway; hoarse voice or cry; wheezing, coughing; sneezing) reported <2 h after ingestion; delayed symptoms are those (worsening of eczema; itching; diarrhoea; constipation; abdominal pain; blood or mucus in stool; vomiting) reported >2 h after ingestion. RN, registered nurse.

**Table 3:** Skin prick test reagents and thresholds

Reagents:
Milk = commercial cow’s milk extract
Egg = commercial egg white extract
Wheat = commercial wheat extract
Soya = commercial soya extract
Plus positive (histamine) and negative (saline) controls
SPT wheal size cutoffs were:
<3 mm = negative
≥3 mm = sensitized

- Introduce/continue to include in diet as normal.
- Home dietary trial of inclusion or exclusion: supported with written information, parents were asked over 2–6 weeks to either include the study food or exclude the study food and then reintroduce it.
- Exclude from diet until OFC: OFCs were offered when the safety of ingesting study food at home was uncertain, deferred when necessary until solid food had been introduced.
- Exclude until review: participants judged to have immediate hypersensitivity to the study food were advised to exclude the food and their GP asked to refer them to an allergist.

Dietary advice pertained to the child only, not breastfeeding mothers.

All dietary advice was supported by researchers trained by the study dietitian, providing elimination advice based on the Food Allergy Specialist Group of the British Dietetic Association. If appropriate, international Milk Allergy in Primary Care milk or British Society for Allergy and Clinical Immunoloy egg ladders were used for the reintroduction of these allergens. At baseline, adherence to dietary advice was not otherwise monitored or enhanced beyond usual care. Any participants excluding foods long-term were offered a consultation with a study dietitian (phone or video call) to ensure dietary needs were met.

An allergy panel (GP, consultant paediatric allergist and dietitian) oversaw the advice being given and were available for guidance throughout the study, for any questions related to eligibility and the interpretation of the SPTs or dietary history that were not covered by the protocol or study working instructions. In case of any discrepancy between the researcher, flowchart and panel, the panel’s opinion prevailed.

### Outcomes

Table 2 shows what data were collected and when. RECap for AtoPic eczema (RECAP), POEM, Peak Pruritis Numerical Rating Scale (PP-NRS), EASI and Infant Dermatitis Quality of Life (IDQOL) are the Harmonising Outcomes Measures for Eczema (HOME)-recommended core outcomes for capturing eczema control, symptoms, itch intensity, signs and disease-specific quality of life, respectively.

The primary outcome is RECAP, collected every 4 weeks for 24 weeks.^17^ RECAP is a seven-item parent-reported measure of eczema control, which has good validity, reliability and responsiveness to change.^18–20^ Each question is scored from 0 to 4 (total score 0–28), where higher scores indicated worse control.

### Participant follow-up and incentives

Participants took part in the trial for 9 months. Families received a £10 voucher at baseline and at the 24-week visit, and were sent regular newsletters.

### Sample size

Our sample size of 493 was based on estimates of a baseline RECAP SD of 6.5 and a baseline-to-24-week correlation of 0.4. If 30% of children in the intervention arm are test-positive and receive dietary advice, and their RECAP score 6.5 units lower than the controls, the overall mean difference between groups will be 1.95 units. To detect this difference with 90% power at a 5% significance level, accounting for 20% lost to follow-up, a sample size of 493 children is required. This provides sufficient power even if 5% of control children seek allergy advice, provided loss to follow-up remains below 10%.

### Masking

Masking status is summarized in Table 4. It was not possible to mask patients and clinicians; therefore, unmasking procedures were not required.

**Table 4:** Masking status of different groups involved in trial delivery

Group	Masking status
Participants^a^ and clinicians	Unmasked
Researchers conducting baseline visits and 24-week visits	Researchers conducting baseline visits cannot be masked to allocation. However, baseline data (including EASI) is collected prior to randomization and where possible, the 24-week visits are conducted by different researchers who are masked to allocation
Trial Manager, Chief/Principal Investigators and allergy panel	Unmasked on a case-by-case basis for operational and safety reasons
Junior statistician	Unmasked to provide data by allocation to the Data Monitoring Committee in closed meetings
Trial Management Group	Masked

EASI, Eczema Area Severity Index. ^a^Parents were asked not to disclose allocation to the research team unless explicitly permitted.

### Statistical methods

Analysis and presentation of the trial data will be in accordance with the CONSORT^21^ and CONSORT PRO^22^ guidelines. A full analysis plan will be made publicly available prior to the analysis. Baseline patient characteristics will be compared using descriptive statistics. Meaningful differences between the groups at baseline will inform any subsequent sensitivity analyses, adjusting for imbalances.

The primary statistical analyses will be conducted on an intention-to-treat principle. The primary analysis for the RECAP score will use a multilevel mixed linear model framework with observations over time for weeks 4 to 24 nested within participants, adjusting for variables used in the randomization.

Secondary outcomes analyses will be done using a multilevel linear model framework for RECAP and POEM scores over weeks 4 to 36, consistent with the primary analysis. Binary outcomes assessed every 4 weeks will be analysed using generalized estimating equations with logistic link. Linear regression models will be applied to IDQOL, Generalised Anxiety Disorder 7 (GAD-7), weight-for-age, stature-for-age and weight-for-stature at week 24 and 36, with adjustment for baseline values. EASI scores at week 24 will also be analysed using linear regression, adjusting for baseline. All models will adjust for variables used in the randomization.

Descriptive analysis of safety endpoints will be presented according to randomized group. Sensitivity analyses of the primary analysis will adjust for any baseline variables showing notable imbalance and examine the impact of missing data, with the approach to handling missingness determined by its nature and pattern. Prespecified subgroup analyses will explore difference in intervention effect based on baseline eczema severity, type of dietary advice (e.g. exclusions based on SPTs vs. delayed symptoms), and who delivered the intervention (university or CRN-based staff). A ‘per protocol’ analysis will be conducted, restricted to participants who fully complied with dietary advice.

### Monitoring and auditing

We regularly monitored the characteristics of screened, referred and randomized patients; dietary advice processes and OFCs; participant retention; changes in consent/permission; data completeness; and adverse events. Two committees, independent of the funder, meet at least every 12 months and monitor the study progress, to ensure it is conducted accordance with good research practice. They comprise:

- Data monitoring – paediatrician, statistician and dietitian.
- Trial steering – academic GP, statistician, health economist, expert in evaluating complex interventions and parent representative.

A copy of their charter can be found in the Supporting Information.

No interim analyses are planned.

The University Hospitals Bristol and Weston National Health Service (NHS) Foundation Trust monitor the study on behalf of the Sponsor. All trial-related documents are available for review by sponsor, Research Ethics Committee and relevant regulatory bodies.

The trial may be prematurely discontinued by the Sponsor, Chief Investigator or Funder due to poor recruitment, new safety information or for other reasons given by the Data Monitoring, Trial Steering or Ethics committees.

### Safety

The trial and intervention are designed to minimize participant risk.

- If concerns arise regarding faltering growth (as defined by the National Institute for Health and Care Excellence),^23,24^ the participant GP will be informed and parents advised to follow this up with them.
- SPTs are widely used and safe. The risk of a systemic allergic reaction is 1 in 10 000, with anaphylaxis^25^ estimated to be 1 in 1 million, comparable to routine immunization.^26^ All tests take place on practice premises with medical support.
- Home dietary trials are only recommended when testing confirms no immediate-type reactions. Short-term food exclusion (4–6 weeks) are standard practice and unlikely to result in loss of tolerance.
- OFC, considered the gold standard for diagnosing IgE-mediated food allergy, are safe and conducted in NHS day-care facilities by trained staff.^27^

Site teams are responsible for identifying and documenting in patient records any adverse events affecting participants during their time in the study. All adverse events will be assessed by a local investigator (or a clinician with delegated study responsibilities) to determine relatedness and seriousness:

- Nonserious events that may be related to a trial procedure or intervention will be recorded through the participant’s case report form.
- Serious events will additionally be recorded in a summary log. If the event is determined to be related to the SPT or the OFC, and no further action is required.
- Any unexpected reactions will be reported to the sponsor.

All serious adverse events and adverse reactions will be followed up by the site until the event resolves, or an outcome has been reached.

### Sponsorship and funding

The study is sponsored by the University of Bristol (research-governance@bristol.ac.uk). The sponsor ensures the study complies with all contractual, legal, insurance, financial and regulatory obligations.

This study is funded by the NIHR Health Technology Assessment programme (NIHR133464). The views expressed are those of the author(s) and not necessarily those of the NIHR or the Department of Health and Social Care.

The sponsor and funder had input into the design and conduct of the study but not its analysis or reporting.

## Results

### Process evaluation

The aim of the process evaluation is to assess intervention processes, fidelity, mechanisms of action, participant and staff experiences, and contextual influences. Qualitative and quantitative data will be triangulated to explore mechanisms of action and to inform interpretation of trial outcomes, future intervention refinement and implemented strategies.

### Recruitment and consent

We sought to conduct in-depth interviews with ∼30 parents, ∼15 GPs allied healthcare professionals or commissioners, and ∼15 researchers or practice nurses, sampled by centre, role and experience.

Parents from the intervention and standard care groups who consented at baseline were purposively sampled to ensure diversity in health literacy, ethnicity, social context, eczema severity, allergy status and region. Verbal consent was confirmed and audio-recorded prior to interviews, following standardized procedures. Parents and health professionals were offered £20 and £45, respectively.

### Individual interviews

Interviews were conducted remotely via phone or video call, lasting 30–60 min, using open and focused questions to explore participants’ experience of the intervention. These interviews were initiated during the internal pilot phase to help inform and refine the intervention.

### Observation of researcher training and trial delivery

Researcher training sessions and selected study appointments were recorded and/or observed, with verbal consent obtained and recorded. Fidelity will be assessed using a bespoke checklist.

### Analysis

Transcripts will be analysed using inductive reflexive thematic ana­lysis^28^ to explore perceptions of the intervention and trial processes.

### Economic evaluation

The aim of the economic evaluation is to compare the costs and consequences of test-guided dietary advice vs. standard care for eczema management, primarily from the NHS perspective, with secondary analyses including non-NHS costs at 36 weeks.

### Identification and measurement of resources

NHS resource-use data were collected from electronic medical records and parent reports at 12, 24 and 36 weeks. Non-NHS resource use, including over-the-counter medications, personal expenses, private treatments and time off work or nursery is captured via parent-completed questionnaires.^29^

### Valuation of resources

NHS resources will be valued using standard sources such as the Unit Costs of Health and Social Care, National Schedule of NHS Costs and British National Formulary/Prescription Cost Analysis.^30,31^ Parent costs will be reported directly and time off work will be valued using the Annual Survey of Hours and Earnings.^32^ Nursery absence will be reported in physical units, valued using the best available method, subject to sensitivity analysis.

### Outcomes

Outcomes will include quality-adjusted life years (QALYs) ­estimated using the Child Health Utility 9D scale (CHU-9D) for ­children and EuroQol-5 Dimension (EQ-5D-5L) for parents, and care­giver burden estimated using CarerQol for parents.

### Analysis

A detailed health economic analysis plan will be made publicly available prior to the analysis. Cost–consequences analysis will relate costs to a range of outcomes, while cost–utility analysis will compare QALYs to NHS costs. Uncertainty will be addressed by bootstrapping, plotting cost-effectiveness acceptability curves, and in sensitivity analyses.

### Genetic substudy

To identify subgroups who may preferentially benefit from food allergy testing, parents were asked about family history of atopy and permission sought for collection of a saliva sample for DNA extraction from the child, for analysis of known genetic risk variants.

Samples were collected by a researcher at the baseline or week 24 appointment and sent in batches to the University of Edinburgh for DNA extraction and single nucleotide polymorphism genotyping. The four most prevalent loss-of-function mutations in *FLG* (R501X, 2282del4, S3247X and R2447X) and rs2212434 on chromosome 11q13.5 will be genotyped using Tagman allelic discrimination as previously reported.^33^

### Ethics and dissemination

#### Protocol amendments

The sponsor can make a nonsubstantial amendment at any time during a trial. Substantial amendments follow Health Research Authority guidance, with sponsorship confirmed before implementation.

#### Consent

Full and ‘Easy Read’ versions of the Participant Information Leaflet were sent to interested families and researchers explained the study, checked understanding and answered any questions. Written consent was received remotely from the child’s parent, electronically via REDCap or on paper,^34^ before any study specific procedures.

Details of all approached participants, including reasons for nonparticipation were recorded in the study case report forms. Parents can withdraw their child from the trial at any time. While patients are not required to provide reasons for their decision, any volunteered reason is documented.

#### Confidentiality

Information from participants is only identifiable via a unique study identification number. Participant data are securely stored on University of Bristol servers and in locked cabinets. Personal data will not be kept for longer than necessary and only for the purpose for which it was collected. Research data will be kept retained until the youngest participant’s twenty-fifth birthday, when all records will be confidentially destroyed.

Data captured on an encrypted audio-recorder were transferred to a University of Bristol file store as soon as possible. Audio recordings were transcribed by university approved transcription services. Transcripts were labelled with a study-assigned participant number and edited to ensure anonymity of participants.

#### Ancillary and post-trial care

Participants receive continuing care as determined clinically appropriate by their attending clinician. This may include food allergy tests.

#### Dissemination policy

A detailed plan for disseminating the trial results will be developed by the trial management group but will include updating the trial registry with, and sending to participating GP surgeries and participants, a summary of findings.

### Patient and public involvement

The questions ‘What role might food allergy tests play in treating eczema?’ and ‘What is the role of [exclusion] diets in treating eczema?’ were identified by as a research priority for patients in a James Lind Alliance exercise.^35^

In the feasibility study, the Trial of Eczema allergy Screening Tests (TEST),^36^ we conducted an online survey of parents of children with eczema, met three times with a group of parents of children with eczema and had lay representation on the trial management group and trial oversight committee. Patient and Public Involvement (PPI) members reviewed and commented on the trial processes and patient-facing documents, many of which have been included in TIGER. We also presented and discussed the findings with patients and stakeholders at a meeting of a national eczema charity (Eczema Outreach Support) and public engagement events.

In preparation for TIGER, we undertook three meetings over 8 months with parents of children with eczema. Members read and provided feedback on a document that set out the need for, and challenges of conducting, a clinical trial; and discussed the research question, what foods to test, how to deliver dietary advice and whether to offer a deferred allergy test to the comparator group. They also shared their thoughts on the acceptability of the proposed changes from the feasibility trial, what they felt the primary outcome should be and how they would feel about their infant having genetic testing as part of a trial.

We have continued to seek meaningful engagement with patients and the public at all stages of the study. Our PPI co­applicant is a parent of a young child with eczema and food allergies. She attends and represents patient/carer perspectives at trial management meetings. She is also a member of our Parent Advisory Group, with whom we meet every 6–12 months. Members have commented on the protocol and design of study materials, helping ensure all patient-facing materials are as appealing and simple to understand as possible; and during recruitment reviewed progress and discussed problems/helped us identify possible solutions. At the end of the study, we will invite them to discuss the implications of the findings and plan pathways to impact.

## Discussion

Trial design and delivery are pragmatic. In terms of eliminating potential allergens from the diet, we have adopted an effectiveness real-life approach (i.e. primarily leaflet-based dietary advice), rather than an efficacy approach (e.g. intensive dietitian input to eliminate all possible dietary allergens). We know that early introduction of allergenic foods reduces the risk of IgE-mediated food allergy, so we favour an inclusive approach: dietary trials of exclusion were limited for 2–4 weeks, a period unlikely to risk loss of oral tolerance and development of immediate-type allergy.

The dietary advice flowchart is based on published guidelines,^37,38^ the Barrier Enhancement for Eczema Prevention (BEEP) study,^39^ the TEST feasibility study^40^ and a consensus exercise.^41^ The latest version of the trial protocol and statistical analysis plan can be accessed via the study website (www.bristol.ac.uk/tiger-study).

We chose SPTs over blood (IgE specific) because concordance between these tests is good,^42^ and SPTs are easier to undertake in primary care and less invasive than blood tests. We chose the four study foods of cow’s milk, hen’s egg, wheat and soya because parents are commonly concerned about them and exclude them without professional advice;^7,43^ and hen’s egg and cow’s milk are the most common cause of food allergies in young children.

At the end of the study, we will have a better understanding of what role, if any, food allergy tests have in the management of children with eczema.

## Supplementary Material

vzag085_Supplementary_Data

## Data Availability

Direct access will be granted to authorized representatives from the sponsor, host institution and the regulatory authorities to permit trial-related monitoring, audits and inspections, in line with participant consent. Data will be kept anonymous on a research data storage facility. Requests for access to data must be via a written confidentiality and data sharing agreement.
