# Supplementary material for: Trial of food allergy IgE tests for Eczema Relief (TIGER): study protocol
Source: Skin Health Dis. 2026 Jul 3;6(5):722–33. doi: 10.1093/skinhd/vzag085 (PMC13623428; doi:10.1093/skinhd/vzag085)
Supplement: Supplementary file 1 [file vzag085_supplementary_data.zip › TIGER Data Monitoring Committee Charter v2.0_13OCT2022_ALL_UPDATED 10SEP2024.pdf]

BRISTOL TRIALS CENTRE

University of BRISTOL

TIGER
Trial of food allergy (IgE) tests for eczema relief

Data Monitoring Committee Charter

	NAME	TITLE	SIGNATURE	DATE
Trial CI	Matthew Ridd	Professor of Primary Health Care		18.10.22
Chair of DMC	Nicola Jay	Consultant Paediatrician - Allergy		18.10.22
Member of DMC	Ly-Mee Yu	Associate Professor		18 Oct 2022
Member of DMC	Kate Grimshaw	Allergy Specialist Dietitian		18 Oct 2022
Member of DMC	Nick Makwana	Paediatric Allergist		15 Oct 2024

Content	Details of DMC
1. Introduction	
Name (& Sponsor's ID) of trial	Trial of IgE tests for eczema relief (TIGER): individually randomised controlled trial of test-guided dietary advice for children with eczema, with internal pilot and nested economic and process evaluations<br><br>NIHR HTA: NIHR133464<br><br>REC Ref. Number: (*TBC*)<br><br>Sponsors Ref: University of Bristol, 2022-585
Objectives of trial, including interventions being investigated	The objectives of this study are:<br>▪ To determine the clinical effectiveness of test-guided dietary advice versus usual care, for the management of eczema.<br>▪ To evaluate the cost effectiveness of test-guided dietary advice in children with eczema.<br>▪ To assess adherence to, and safety of, test-guided dietary advice in children with eczema.<br>▪ To identify sub-groups who may preferentially benefit from food allergy testing.
Outline of scope of Charter	The purpose of this document is to describe the membership, roles, and responsibilities of the Data Monitoring and Ethics Committee (DMC) for the TIGER trial, including the timing of meetings, methods of providing information to and from the DMC, frequency and format of meetings, statistical issues and relationships with other trial committees.
Facilitation	The Trial Manager at the Bristol Medical School will be nominated as a Facilitator for the trial. The Trial Manager will be responsible for the organisation of meetings.
Content	Details of DMC
2. Roles and responsibilities	
Aims of the DMC	To safeguard the interests of the trial's participants, potential participants, investigators and sponsor; to assess the safety

	and efficacy of the trial's interventions, and to monitor the trial's overall conduct, and protect its validity and credibility.
Terms of reference	The DMC should receive and review reports on the data accruing to this trial and make recommendations on the conduct of the trial to the TSC.
Specific roles of DMC	To undertake interim review of the trial's progress in the following ways:<br>• receive and review reports on data quality, including completeness (thereby encouraging collection of high-quality data)<br>• receive and review reports on recruitment and losses to follow-up<br>• receive and review reports on compliance with the protocol by participants and investigators<br>• receive and review reports on any agreed interim analyses, and so consider evidence for treatment differences in the main efficacy and safety outcome measures – and thus recommending action when/whether the main trial question has been answered<br>• receive and review reports on the evidence for treatment harm e.g. toxicity, SAEs, deaths<br>• recommend whether the trial should continue to recruit or follow-up [see section on decision-making]<br>• recommend any major changes to the protocol, where necessary (e.g. changes to the recruitment procedures, inclusion criteria, endpoints, data collection, etc)<br>• advise on and/or endorse any major protocol modifications suggested by investigators or sponsors (e.g. changes to the inclusion criteria, endpoints, data collection, etc)<br>• monitor the planned sample size with regards (i) *a priori* assumptions about the control arm outcome and (ii) emerging differences in clinically relevant subgroups<br>• suggest additional data analyses<br>• assess the impact and relevance of any external evidence provided<br>• monitor actions taken in response to previous DMC recommendations<br>• consider the ethical implications of any recommendations made by the DMC

	The DMC should **not** have a role in increasing or decreasing the planned sample size as it is not blind to the current results of the trial. (See decision making)
Content	Details of DMC
3. Before or early in the Trial	
Invitations to join the DMC	All potential DMC members will have had sight of the protocol/outline before agreeing to join the committee. Before recruitment begins the trial will have undergone review by the Sponsor and Funder (e.g. peer review for public sector trials), scrutiny by other trial committees and a research ethics committee. Therefore, if a potential DMC member has major reservations about the trial (e.g. the protocol, the logistics, ethical concerns) they should report these to the Bristol Trials Centre (BTC) and may decide not to accept the invitation to join. DMC members should be independent and constructively critical of the ongoing trial, but also supportive of aims and methods of the trial.
The first DMC meeting	The DMC should meet early in the trial, preferably in person. The timing should be within one year of commencing recruitment, or before 10% of the planned events have been reported; whichever is sooner. The aim of the first meeting would be to discuss and agree the working of the DMC: specifically, to agree the charter, and the format of reports to future meetings.<br><br>The study protocol will indicate if the DMC will be asked to review interim analyses. A detailed plan for interim analyses (i.e. unblinded analyses of outcome data which inform recommendations about the subsequent conduct of the trial) will be agreed prior to any such analyses and described in the Statistical Analysis Plan for the trial.<br><br>A joint face-to-face "kick-off" meeting between the TSC and DMC can work well, particularly in establishing how the two committees will communicate.
Regulatory issues	None raised.
Any other issues specific to the intervention under study	None raised.
Whether members of the DMC will have a contract	DMC members will not be asked to formally sign a contract but should formally register their agreement to join the group by confirming (1) that they agree to be a member of the DMC and (2) that they agree with the contents of this document. Any potential competing interests should be declared at the same time. Members should complete and return the form in Annexe 1.

Content	Details of DMC
4. Composition	
Membership and size of the DMC	The membership will consist of 3 individuals that include at least one clinician, and at least one statistician. Members have been chosen because they are experienced in trials and/or the disease area.<br>The chairperson and members of the DMC have been recommended by the TMG and approved by the TSC/funder.<br>The members should be independent of the trial (independent as defined in table 1 of Annexe 1). Members should not serve on DMCs of similar, concurrently active trials as this could compromise the independence of the trial and possibly the confidentiality of the results of the individual trials. Any competing interests, both real and potential, should be declared within the completed form in Annexe 1.<br>Note: "Competing interests" is the preferred term over "conflicts of interests" because it avoids any implication of wrongdoing and may encourage greater co-operation in reporting interests.
The responsibilities of the DMC Statistician	The DMC statistician is to provide independent statistical expertise and to further guide the other DMC members through the report prepared by the trial team.
The responsibilities of the trial statistician	The trial statistician is responsible for the production of the report to the DMC, will guide the DMC through the report, may participate in DMC discussions, will comment on minutes, and may have to take notes on some occasions.
The responsibilities of the Chief Investigator (CI) and other members of the TMG	The CI may be asked and should be available to attend open sessions of the DMC meeting. The other TMG members will not usually be expected to attend but can attend open sessions when necessary (see Organisation of meetings).
Content	Details of DMC
5. Relationships	
Relationships with Chief Investigators, other trial committees (e.g. TMG and TSC), Sponsor/Funder and regulatory bodies	The responsibilities of each trial committee are detailed in the protocol and the respective Terms of Reference. Relationships between these groups are summarised in Figure 1.
Advisory and executive bodies	The TSC is the executive body of the trial. The DMC is advisory to the TSC.
Payments to DMC members	Members will be reimbursed for reasonable travel costs (standard rail fares and if travelling by car, mileage up to the

	amount of the equivalent rail fare) and other reasonable costs incurred. No other payments or rewards would be given to professional members.
The need for DMC members to disclose information about any real or potential competing interests	Competing interests should be disclosed both at the point of joining the DMC, and as they arise during the lifetime of the DMC e.g. by having this declaration as an agenda item for DMC meetings. These are not restricted to financial matters – involvement in other trials or intellectual investment could be relevant. Although members may well be able to act objectively despite such connections, complete disclosure enhances credibility (see Annexe 1). DMC members should not use interim results to inform trading in stock of companies with related products.
Content	Details of DMC
6. Organisation of meetings	
Expected frequency of DMC meetings	The DMC will meet at least annually, face to face or by teleconference. At the request of the DMC and/or Trial Office, interim meetings, in person or by teleconference, will be organised. Major trial issues may need to be dealt with between meetings, by phone or by email. DMC members should be prepared for such eventualities.
Attendance of DMC members at meetings	The first meeting will be face to face It is recommended that all subsequent meetings should be face to face too, teleconferences as a second option. The CI should try to attend in person if the DMC requests their presence.
How DMC meetings will be organised, especially regarding open and closed sessions, including who will be present in each session	<u>Open session</u>: Attended by those at the closed session, plus the CI(s), and perhaps also representatives of the sponsor, funder, or regulator, as relevant, and BTC representative. Those attending only the open session may attend in person or, if appropriate by phone.<br><br><u>Closed session</u>: Only DMC members and the trial statistician should be present in closed sessions (although the DMC should be able to ask the trial statistician to leave, also). Sessions attended only by DMC members may be referred to as executive sessions.<br><br>The format will be:<br><br>1 Open session: Discussion with other attendees on any matters arising from the closed session (this assumes that others will have read the “open” report in advance).<br><br>2 Closed session: All parts of the report are discussed, and DMC discussion (± trial statistician at discretion of DMC).
Content	Details of DMC

7. Trial documentation and procedures to ensure confidentiality and proper communication	
Intended content of material to be available in open sessions	Accumulating information relating to recruitment and data quality e.g. data return rates, treatment compliance. Toxicity details based on pooled data may be presented, although this may be presented by arm if toxicity is not the primary outcome measure. Total numbers of events for the primary outcome measure and other outcome measures.
Intended content of material to be available in closed sessions	In addition to all the material available in the open session, the closed session material would usually include efficacy and safety data by treatment group. The DMC will not be blinded to the identity of the treatment arms.
Who will see the accumulating data and interim analysis by randomised group	The accumulating trial data by arm and interim analyses will be confidential. These will be viewed only by the DMC (closed sessions). The TSC will not be routinely privy to these interim reports. The DMC will make recommendations to the TSC based on the interim data. DMC members must **not** share confidential information with people outside the DMC, including the CI.
Responsibility for identifying and circulating external evidence (e.g. from other trials/ systematic reviews)	Identification and circulation of external evidence (e.g. from other trials/systematic reviews) is a responsibility of the TMG.
To whom the DMC will communicate the decisions that are made	The DMC will report its recommendations in writing to the Trial Steering Committee. If the trial is to continue largely unchanged, then it is often useful for the report from the DMC to include a summary paragraph suitable for trial promotion purposes.
Whether reports to the DMC be available before the meeting or only at/during the meeting	It is planned that the DMC will receive the report from the BTC at least 2 weeks before any meetings.
What will happen to the papers after the meeting	The DMC members will store their copies of the meeting papers securely after each meeting so they can check future reports against them. All copies of trial reports and papers held by DMC members will be destroyed at the end of the trial (end of trial as defined in the Protocol).
Content	Details of DMC
8. Decision making	
Patient Safety	The safety and well-being of the trial participants are the most important considerations and should prevail over the interests of science and society.
What decisions/recommendations will be open to the DMC	The possible recommendations are numerous and could include:

	• No action needed; trial continues as planned<br>• Early stopping due, for example, to clear benefit or harm of an intervention or external evidence (this should generally involve a recommendation from the DMC to unblind the TSC to these data)<br>• Stopping recruitment within a subgroup** (this should generally involve a recommendation from the DMC to unblind the TSC to these data)<br>• Advising and/or proposing protocol changes<br>• Censuring centres for poor recruitment/poor data quality<br>**Note**: the DMC will not advise on changes to the target sample size that are based on emerging differences between trial arms.<br>** Considerable care should be taken if this is not a pre-specified subgroup
Early stopping of recruitment	A recommendation to discontinue recruitment, in all patients or in selected subgroups, will be made only if the result is likely to convince a broad range of clinicians, including those supporting the trial and the general clinical community.
How decisions or recommendations will be reached within the DMC	Every effort should be made to achieve consensus. The role of the Chair is to summarise discussions and encourage consensus; therefore, it is usually best for the Chair to give their own opinion last.<br>If the DMC cannot achieve consensus, a vote should be taken, although details of the vote should not be routinely included in the report to the TSC, as it may inappropriately convey information about the state of the trial data.
When the DMC is quorate for decision-making	Effort should be made for all members to attend. The BTC trial team will try to ensure that a date is chosen to enable this. Members who cannot attend in person should be encouraged to attend by teleconference. If, at short notice, any DMC members cannot attend at all then the DMC may still meet if at least one statistician and one clinician, including the Chair (unless otherwise agreed), will be present. If the DMC is considering recommending major action after such a meeting the DMC Chair should talk with the absent members as soon as possible after the meeting as possible to check they agree. If they do not, a further teleconference should be arranged with the full DMC.
Content	Details of DMC
9. Reporting	

To whom will the DMC report their Recommendations/decisions, and in what form	The DMC should report in writing to the Trial Steering Committee, usually within 3 weeks after the meeting. This should be copied to the trial statistician and may be sent via the BTC. Unless the DMC is recommending that the trial protocol be changed in some way, the letter to the TSC should not usually reveal any confidential information. Additionally, the letter should be copied to the CI, although their copies should have any confidential information (i.e. comments on data by group on the main outcome measures) removed.
Whether minutes of the meeting be made and, if so, by whom and where they will be kept	Minutes from the meeting need not be detailed. A summary of the main points discussed with a list of clearly marked action points should be sufficient. It is recommended that separate minutes of open and closed session be made if the meeting is run in this format. The minutes for each session should be made only by someone who attends that session. This should be agreed at the start of the meeting. All members of the DMC should see and comment on the minutes. The DMC chair will be responsible for signing off all minutes.
What will be done if there is disagreement between the DMC and other trial committees	The TSC is the executive body for the trial. However, the TSC should have good reason before deciding not to accept recommendations from the DMC. If there are serious problems or concerns with the TSC decision following a DMC recommendation, a joint meeting of the TSC and DMC should be held. The information to be shown would depend upon the action proposed and each committee’s concerns. Depending on the reason for the disagreement confidential data may have to be revealed to all or some of those attending such a meeting: this would be minimised where possible. The meeting would be Chaired by a senior member of the BTC or an external expert who is not directly involved with the trial.
Content	Details of DMC
10. After the trial	
Publication of results	The TSC will oversee the timely analysis, writing up and publication of the main trial results. The DMC members will have the opportunity to read and comment on the proposed main publications of trial data prior to submission. This review may be concurrent to that of the trial investigators and TSC.
The information about the DMC that will be included in published trial reports	DMC members will be named, and their affiliations listed in the main report, unless they explicitly request otherwise. A brief summary of the timings and conclusions of DMC meetings should be included in the body of this paper.

Whether the DMC will have the opportunity to approve publications, especially with respect to reporting of any DMC recommendation regarding termination of a trial	The DMC members should be given 2 weeks to read and comment on any draft publications that report outcome measures and/or details of the DMC. This may be done simultaneously to other groups reviewing the draft manuscript (e.g. Trial Steering Committee, trial investigators).
Any constraints on DMC members divulging information about their deliberations after the trial has been published	The DMC should not discuss confidential information to what they have been privy to as a result of their involvement in the trial until 12 months after the primary trial results have been published. They should not trade in stock of companies affected by the trial until the results are public knowledge.

Figure 1: Relationships of Trial Committees

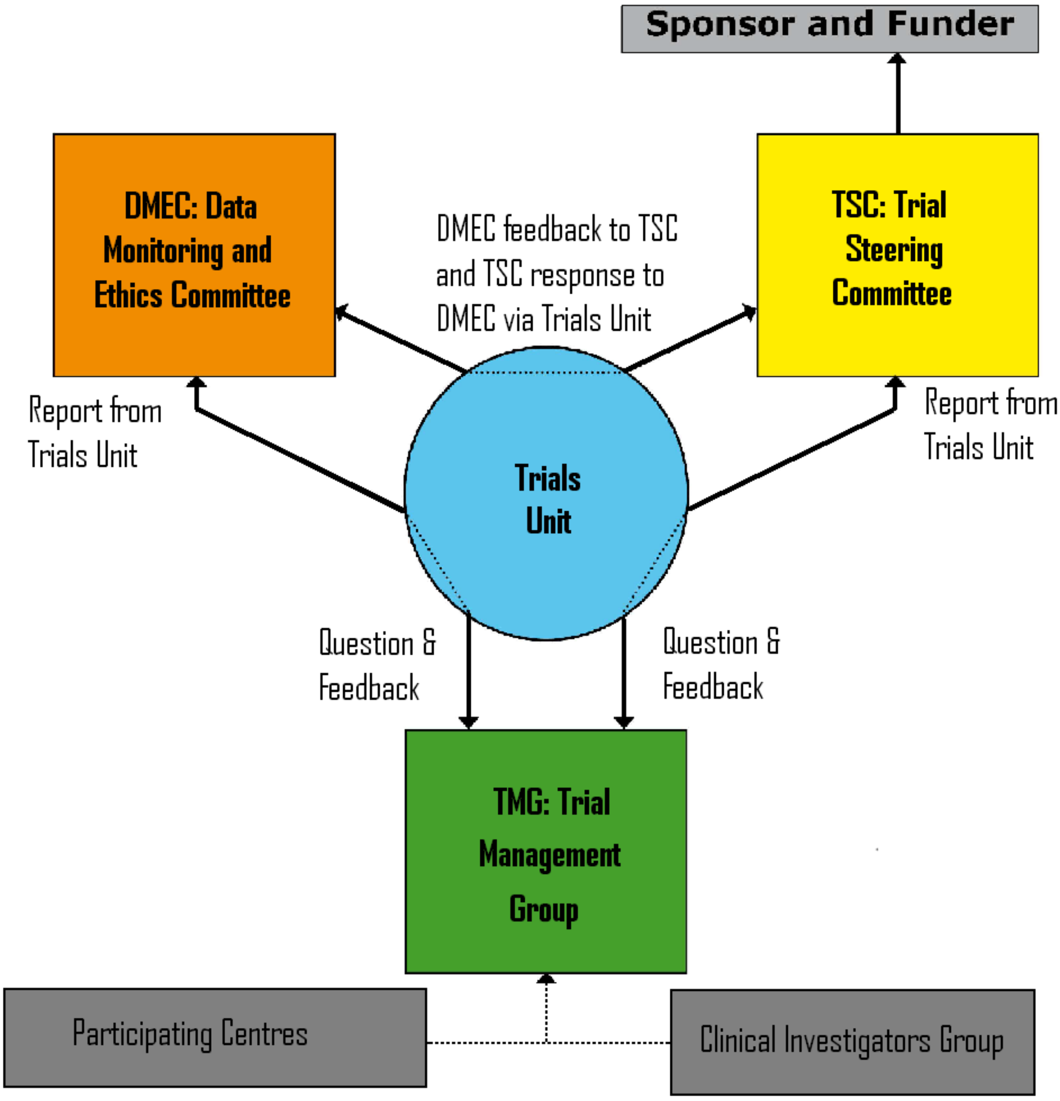

Please note that "Trials Unit" can be replaced by "Trial Manager" where appropriate.

Annexe 1: Agreement and competing interests form for Data Monitoring and Ethics Committee members

TIGER Study (NIHR133464): Agreement to join the Data Monitoring and Ethics Committee as an independent member and disclosure of potential competing interests

Please complete the following document and return to the DMC Facilitator.

(please **initial** box to agree)

☐ I have read and understood the DMC Terms of Reference, Version 2.0, 13 October 2022

☐ I agree to join the Data Monitoring and Ethics Committee for this trial as an independent member

☐ I agree to treat all sensitive trial data and discussions confidentially

The avoidance of any perception that independent members of a DMC may be biased in some fashion is important for the credibility of the decisions made by the DMC and for the integrity of the trial.

Potential competing interests should be disclosed via the BTC. In many cases simple disclosure up front should be sufficient. Otherwise, the (potential) DMC member should remove the conflict or stop participating in the DMC. **Table 1** lists potential competing interests.

☐ **No**, I have no competing interests to declare

☐ **Yes**, I have competing interests to declare (please detail below)

Please provide details of any competing interests:

Name: ____

Signed:____Date:_________________________

Table 1: Potential competing interests for independent members

Consulting arrangements with the Sponsor/Funder
Frequent speaking engagements on behalf of the intervention
Career tied up in a product or technique assessed by trial
Hands-on participation in the trial
Involvement in the running of the trial
Emotional involvement in the trial
Intellectual conflict e.g. strong prior belief in the trial's experimental arm
Involvement in the writing up of the main trial results in the form of authorship
