# Supplementary material for: Trial of food allergy IgE tests for Eczema Relief (TIGER): study protocol
Source: Skin Health Dis. 2026 Jul 3;6(5):722–33. doi: 10.1093/skinhd/vzag085 (PMC13623428; doi:10.1093/skinhd/vzag085)
Supplement: Supplementary file 1 [file vzag085_supplementary_data.zip › TIGER Good Eczema Care Leaflet.pdf]

University of BRISTOL

BRISTOL TRIALS CENTRE

TIGER
Trial of food allergy (IgE) tests for eczema relief

You have been given this leaflet because you are taking part in the TIGER study. It summarises good advice on how to look after your child's skin.

About eczema (atopic eczema/dermatitis)

Eczema is a long-term condition. It can improve or clear completely in some children as they get older. The cause of eczema is unknown. Symptoms tend to come and go: there may be times with little or no symptoms, and "flare-ups" when the skin is more inflamed, sore or itchy than usual. Inflamed skin looks red on lighter skin, and darker brown, purple or grey on darker skin.

What can I do to help my child's eczema?

Eczema cannot be cured but it can be treated effectively with just two treatments used well – "getting control" with flare control treatments and "keeping control" with moisturisers.

👍 Always wash your hands before you apply any creams to your child's skin.

Get control – flare control creams

Flare control creams are used to reduce inflammation and itching during flare-ups. The most common type are topical corticosteroids. Used correctly, they are safe and effective for your child.

Topical corticosteroids come in different strengths, which you can only tell from the information leaflet that comes with the tube (not their "%"). For example, hydrocortisone 1% is mild; clobetasone butyrate (Eumovate) is moderate; betamethasone valerate 0.1% (Betnovate) is strong; and clobetasol proprionate (Dermovate) is very strong. Which ones your health care professional recommends will depend on the severity of your child's eczema and the part of the body affected.

Start using your flare control cream as soon as you spot a flare-up to get control quickly:

- Usually, you only have to apply once a day
- Put on a light covering, just enough to cover the eczema flare area.
- For a mild eczema flare-up, it usually takes 5-7 days to get control. For a thicker patch of eczema or more severe flare-up it may take a few weeks.
- Continue to use it two days after the flare-up has cleared, to treat the inflammation under the skin surface
- Leave a gap of at least 20-30 minutes between putting on flare control and moisturising creams (or use them at different times of the day)
- Do not use continuously for longer than 4 weeks without discussing with your doctor

Topical calcineurin inhibitors including pimecrolimus (Elidel) cream and tacrolimus (Protopic) are sometimes prescribed as an alternative to topical corticosteroids, especially on delicate areas.

Keep control – moisturisers

Moisturisers can help with dry skin. Using them every day on your child's skin can help prevent or reduce eczema flare-ups.

They come as lotions, creams, gels, ointments and sprays. Examples include QV lotion, Epimax cream, Myribase gel and Zeroderm ointment. All types are effective, but you might prefer one type to another. Moisturisers are safe and can be used all over the body (but be careful to not get in the eyes).

- Apply liberally and regularly
- Smooth moisturisers into the skin in the same direction the hair grows.
- Stroke it in, as rubbing can make your child itchy.

If your child is having a flare-up, you should use your moisturisers more frequently, but also treat the areas of inflamed skin with a flare control cream or ointment.

Tips:

- Moisturise your child's hands and/or around their mouth before messy activities or eating.
- Many moisturisers can be used as soap substitutes.
- Use a spoon (not your fingers) to get moisturiser out of a pot.
- Avoid naked flames. Clothing and bedding that have been in contact with moisturisers burn more easily, even after having been washed.

Bathing

There is no guidance about how often people with eczema should bath or shower. Go with whatever is comfortable for you and your child.

- Bathe for up to 15 minutes in warm but not hot water. Longer than this can dry the skin out and hot water can trigger itching.
- Don't put anything in the bath that is perfumed or creates bubbles, including shampoo.
- Pat the skin dry with a towel, rather than rubbing dry.
- Apply moisturisers as soon as possible after bathing.
- Moisturisers can make the bath or shower slippery, so clean the bath/shower afterwards.

Avoid triggers

The following can trigger itching and eczema flares:

- Wool and rough synthetic fabrics
- Soaps, shampoos, bubble bath and perfumed products
- Wet/messy play
- Allergens such as house dust mite, pollen, pet fur and moulds

Keep the rooms in your home cool (especially the bedroom) and nails short and clean (using mittens in babies) to reduce the damage from scratching.

If your child's skin suddenly worsens, weeps, crusts or blisters, it could be infected, and your child may need other treatment – seek advice from your doctor.

☹ Remember also that your child's eczema may get better or worse for no obvious reason.

For more information and advice

Go to EczemaCareOnline.org.uk – a free website, created in partnership by people with eczema and NHS experts.
