# Supplementary material for: Trial of food allergy IgE tests for Eczema Relief (TIGER): study protocol
Source: Skin Health Dis. 2026 Jul 3;6(5):722–33. doi: 10.1093/skinhd/vzag085 (PMC13623428; doi:10.1093/skinhd/vzag085)
Supplement: Supplementary file 1 [file vzag085_supplementary_data.zip › TIGER Trial Steering Committee Terms of Reference v1.0 23 SEP 2022.pdf]

TIGER
Trial of food allergy (IgE) tests for eczema relief

Trial Steering Committee Terms of Reference

	NAME	TITLE	SIGNATURE	DATE
Trial CI	Matthew Ridd	Professor of Primary Health Care		
Chair of TSC	Tom Fahey	Professor and Head of Department of General Practice		
Member of TSC	Myka Ransom	Senior Medical Statistician		
Member of TSC	Lazaros Andronis	Reader in Health Economics & NIHR Advanced Career Fellow		
Member of TSC	Rhiannon Evans	Reader in Social Science and Health		
Member of TSC	Sariqa Wagley	PPI Member		

Content	Details of TSC
1. Introduction	
Name (& Sponsor's ID) of trial	Trial of IgE tests for eczema relief (TIGER): individually randomised controlled trial of test-guided dietary advice for children with eczema, with internal pilot and nested economic and process evaluations<br><br>NIHR HTA: NIHR133464<br><br>REC Ref. Number: (*TBC*)<br><br>Sponsors Ref: University of Bristol, 2022-585
Objectives of trial, including interventions being investigated	The objectives of this study are:<br><br>• To determine the clinical effectiveness of test-guided dietary advice versus usual care, for the management of eczema.<br>• To evaluate the cost effectiveness of test-guided dietary advice in children with eczema.<br>• To assess adherence to, and safety of, test-guided dietary advice in children with eczema.<br>• To identify sub-groups who may preferentially benefit from food allergy testing.
Outline of scope of Charter	The purpose of this document is to describe the membership, terms of reference, roles, responsibilities, authority, decision-making and relationships of the Trial Steering Committee (TSC) for this trial, including the timing of meetings, methods of providing information to and from the TSC, frequency and format of meetings and relationships with other trial committees.
Facilitation	The Trial Manager at the Bristol Medical School will be nominated as a Facilitator for the trial. The Trial Manager will be responsible for the organisation of meetings.
Content	Details of TSC
2. Roles and responsibilities	
Aims of the TSC	To act as the oversight body for this trial on behalf of the Sponsor and Funder.
Terms of reference	The role of the TSC is to provide oversight for the trial. It should also provide advice through its independent Chair to the Trial Management Group (TMG), the funder (NIHR Health Technology Assessment (HTA) Programme) and the Bristol Trials Centre (BTC) on all aspects of the trial.
Specific roles of TSC	The specific roles include:<br><br>• provide expert oversight of the trial<br>• maintain confidentiality of all trial information that is not already in the public domain<br>• make decisions as to the future continuation (or

	otherwise) of the trial<br>• monitor recruitment rates and encourage the TMG to develop strategies to deal with any recruitment problems<br>• approve the protocol<br>• review regular reports of the trial from the TMG<br>• receive letters of feedback from the DMC and consider their recommendations<br>• assess the impact and relevance of any accumulating external evidence<br>• monitor completeness of data and comment on strategies from TMG to encourage satisfactory completion in the future<br>• monitor follow-up rates and review strategies from TMG to deal with problems<br>• censure sites that are deviating from the protocol<br>• approve any amendments to the protocol, where appropriate<br>• approve any proposals by the TMG concerning any change to the design of the trial, including additional sub-studies<br>• oversee the timely reporting of trial results<br>• approve/comment on the publication policy<br>• approve/comment on the main trial manuscript<br>• approve external or early internal requests for release of data or subsets of data or samples including clinical data<br>• provide evidence to support any requests for extensions, indicating that all practicable steps have been taken to achieve targets.
Content	Details of TSC
3. Before or early in the Trial	
Whether the TSC will have input into the protocol	All potential TSC members will have had sight of the protocol/outline before agreeing to join the committee. Before recruitment begins the trial will have undergone review by the Sponsor and Funder (e.g. peer review for public sector trials), scrutiny by other trial committees and a research ethics committee. Therefore, if a potential TSC member has major reservations about the trial (e.g. the protocol, the logistics, ethical concerns) they should report these to the BTC and may decide not to accept the invitation to join. TSC members should be independent and constructively critical of the ongoing trial, but also supportive of aims and methods of the trial.
The first TSC meeting	The TSC should meet early in the trial, preferably in person. The timing should be within one year of commencing recruitment, or before 10% of the planned events have been reported; whichever is sooner. The aim of the first meeting would be to discuss and agree the working of the TSC: specifically, to agree the charter, and the format of reports

	for future meetings.<br><br>The study protocol will indicate if the TSC will be asked to review interim analyses. A detailed plan for interim analyses (i.e. unblinded analyses of outcome data which inform recommendations about the subsequent conduct of the trial) will be agreed prior to any such analyses and described in the Statistical Analysis Plan for the trial.<br><br>A joint face-to-face "kick-off" meeting between the TSC and DMC can work well, particularly in establishing how the two committees will communicate.
Regulatory issues	None raised.
Any other issues specific to the intervention under study	None raised.
Whether members of the TSC will have a contract	TSC members will not be asked to formally sign a contract but should formally register their agreement to join the group by confirming (1) that they agree to be a member of the TSC and (2) that they agree with the contents of this document. Any potential competing interests should be declared at the same time. Members should complete and return the form in Annexes 1 or 2. Any observers (attendees who are not members) will sign a confidentiality agreement on the first occasion they attend a meeting (Annexe 3).
Content	Details of TSC
4. Composition	
Membership and size of the TSC	The majority of members of the TSC, including the Chair, should be independent* of the trial. Members have been chosen because they are experienced in trials and/or the disease area.<br><br>The chairperson and members of the TSC have been recommended by the TMG and approved by the TSC/funder.<br><br>Non-independent members will also be part of the TSC.<br><br>The members of the TSC for this trial are:<br><br>1. Independent member: Prof. Tom Fahey (Chair), Professor and Head of Department, Department of General Practice, RCSI University of Medicine and Health Sciences, 123 St. Stephens Green, Dublin 2, Ireland.<br>2. Independent member: Myka Ransom, Senior Medical Statistician, Leeds Institute of Clinical Trials Research, University of Leeds.<br>3. Independent member: Dr Lazaros Andronis, Reader in Health Economics and NIHR Advanced Career Fellow, University of Warwick.<br>4. Independent member: Dr Rhiannon Evans, Reader in

	Social Science and Health, DECIPHer (Centre for Development, Evaluation, Complexity, and Implementation in Public Health Improvement).<br>5. Independent member: Ms Sariqa Wagley, PPI member.<br>6. Non-independent member: Prof Matthew Ridd, Professor of Primary Health Care, Bristol Medical School, University of Bristol.<br><br>(* Independence is defined in Table 1 of Annexe 1)<br><br>Any competing interests, both real and potential, should be declared within the completed form in Annexe 1.<br><br>Note: “Competing interests” is the preferred term over “conflicts of interests” because it avoids any implication of wrongdoing and may encourage greater co-operation in reporting interests.
The responsibilities of the trial team	The trial team will produce a short report on the trial before each meeting of the TSC.
The responsibilities of the Chief Investigator (CI) and other members of the TMG	The CI (and, if appropriate, other TMG members) is an important member of the TSC and no major decisions should be made without their involvement.
The responsibilities of the observers	Additional observers may be in attendance through (parts of) the TSC meetings in order to provide input on behalf of the BTC, the trial’s Sponsor/Funder or to provide specific relevant expertise.
Content	Details of TSC
5. Relationships	
Relationships with Chief Investigators, other trial committees (e.g. TMG and DMC), Sponsor/Funder and regulatory bodies	The responsibilities of each trial committee are detailed in the protocol and in the respective Charters. The relationships between these groups are summarised in Figure 1.
Advisory and executive bodies	The TSC is the executive body of the trial and is delegated the roles in Section 2 by the Sponsor. All substantial issues regarding the trial must go to the TSC for consideration. The Data Monitoring Committee (DMC) is advisory to the TSC.
Payments to TSC members	Members will be reimbursed for reasonable travel costs (standard rail fares and if travelling by car, mileage up to the amount of the equivalent rail fare) and other reasonable costs incurred. No other payments or rewards would be given to professional members. Honoraria will be paid to lay members.
The need for TSC members to disclose information about any real or potential competing interests	Competing interests should be disclosed both at the point of joining the TSC, and as they arise during the lifetime of the TSC e.g. by having this declaration as an agenda item for TSC meetings. These are not restricted to financial matters – involvement in other trials or intellectual investment could be relevant. Although members may well be able to act objectively despite such connections, complete disclosure

	enhances credibility (see Annexe 1). TSC members should not use interim results to inform trading in stock of companies with competing or related products.
Content	Details of TSC
6. Organisation of meetings	
Expected frequency of TSC meetings	The TSC will meet at least annually, face to face or by teleconference. At the request of the TSC and/or Trial Office, interim meetings, in person or by teleconference, will be organised. Major trial issues may need to be dealt with between meetings, by phone or by email. TSC members should be prepared for such eventualities.
Attendance of TSC members at meetings	Every effort will be made to ensure that all members can attend. The Trial office will arrange a date that enables this. The CI would be expected to attend all meetings, especially if major actions are expected. Members who cannot attend in person should be encouraged to participate by teleconference. If, at short notice, any TSC members cannot attend then the TSC may still meet if at least two independent members, plus the Chair (unless otherwise agreed), will be present, plus also a member of the trial team. If the TSC is considering a major action after such a meeting the TSC Chair should communicate with the absent members, including the CI, as soon after the meeting as possible to check they agree. If they do not agree, a further teleconference should be arranged with the full TSC.
How TSC meetings will be organised, especially regarding open and closed sessions, including who will be present in each session	Presence will be usually limited to the TSC members, observers from the Sponsor/Funder, trials unit and the trial manager. Other attendees may be invited for all or part of the meeting by the TSC including the trial statistician. The observers are not members of the TSC but may be invited to provide expert input. Representatives from the funding body and Sponsor should be invited to every TSC; other observers will be at the discretion of the TSC and trial manager but may include members of the TMG other than the CI.
Can TSC members who cannot attend the meeting input	If the report is circulated before the meeting, TSC members who will not be able to attend the meeting may pass comments to the TSC Chair for consideration during the discussions.
What happens to independent members who do not attend meetings	If an independent member does not attend a meeting or provide comments when requested between meetings, it should be ensured that the independent member is available for the next meeting. If an independent member does not attend the next meeting or provide comments when next requested, they should be asked if they wish to remain part of the TSC. If an independent member does not attend a third meeting, strong consideration should be given to replacing this member.
Content	Details of TSC
7. Trial documentation and procedures to ensure confidentiality and proper communication	

Intended content of material to be considered during meetings	A short report will be prepared by the trial team. This will report on recruitment and any matters affecting the trial. Additionally, the material may include a report from the DMC, requests from the TMG or draft publications. No trial outcome measure data will be presented by arm unless explicitly authorised by the DMC. Where relevant, recruitment, compliance with follow-up and adherence may be presented by centre.
Who will see the accumulating data and interim analysis by randomised group	The accumulating trial data by arm and interim analyses will be confidential. These will be viewed only by the DMC. The TSC will not be routinely privy to these interim reports. The DMC will make recommendations to the TSC based on the interim data.
Responsibility for identifying and circulating external evidence (e.g. from other trials/ systematic reviews)	Identification and circulation of external evidence (e.g. from other trials/systematic reviews) is a responsibility of the TMG. The TSC should continue to be made aware of other data that may impact on the trial.
Whether reports to the TSC be available before the meeting or only at/during the meeting	The TSC should receive the report at least 1 week and preferably 2 weeks before any meetings. Different procedures may apply to teleconference meetings.
What will happen to the papers after the meeting	The TSC members will store their copies of the meeting papers securely after each meeting so they can check future reports against them. After the trial has been reported, TSC members will destroy all copies of reports.
Content	Details of TSC
8. Decision making	
Patient Safety	The safety and well-being of the trial participants are the most important considerations and should prevail over the interests of science and society.
What decisions will be open to the TSC	Based on recommendations from the DMC, possible decisions include:<br><br>• No action needed; trial continues as planned<br>• Early stopping due, for example, to clear benefit or harm of an intervention or external evidence (this should generally involve a recommendation from the DMC to unblind the TSC to these data)<br>• Stopping recruitment within a subgroup** (this should generally involve a recommendation from the DMC to unblind the TSC to these data)<br>• Modifying target recruitment, or pre-analysis follow-up, based on any change to the assumptions underlying the original trial sample size calculation (but not on any emerging differences)<br>• Sanctioning and/or proposing protocol changes<br><br>Based on other factors, possible decisions include the decisions above and:<br><br>• Censuring centres for poor recruitment/poor data

	quality<br>• Approving proposed protocol amendments or new trial sub-studies<br>• Approving requests for early release of (subsets of) data<br>• Approving presentation of results during the trial or soon after closure<br>• Approval of strategies to improve recruitment or follow-up<br><br>** Considerable care should be taken if this is not a pre-specified subgroup
The role of formal statistical methods	Formal statistical methods may have been considered by the DMC in making their recommendations to the TSC. These methods are usually used as guidelines rather than absolute rules. This is because they generally only consider one dimension of the trial. The DMC will record reasons for disregarding a stopping guideline in the notes of their meetings and may choose to also note this in their report to the TSC if necessary.
How decisions or recommendations will be reached within the TSC	Every effort should be made to achieve consensus. The role of the Chair is to summarise discussions and encourage consensus; therefore, it is usually best for the Chair to give their own opinion last.<br><br>If the TSC cannot achieve consensus, a vote should be taken. It is important that all the implications (e.g. ethical, statistical, practical, financial) for the trial are considered before any decision is made.
When the TSC is quorate for decision-making	Effort should be made for all members to attend. The trial team will try to ensure that a date is chosen to enable this.<br><br>At least two independent members of the TSC should be present plus the Chair, plus a representative of the trials unit and, if major action is to be considered, the CI.
Content	Details of TSC
9. Reporting	
To whom will the TSC report their recommendations/decisions, and in what form	The TSC will report their decisions (usually within 3 weeks after the meeting) to the TMG who will be responsible for implementing any actions resulting. The TSC may also provide feedback to the DMC and, where appropriate, to the Sponsor and Funder.
Whether minutes of the meeting be made and, if so, by whom and where they will be kept	Minutes from the meeting need not be detailed. A summary of the main points discussed with a list of clearly marked action points should be sufficient. A note should be made of whether potential competing interests have changed for any attendees since the previous meeting.<br><br>The minutes for each session should be made only by

	someone who attends that session. This should be agreed at the start of the meeting. All members of the TSC should see and comment on the minutes. The TSC chair will be responsible for signing off all minutes.
What will be done if there is disagreement between the TSC and other trial committees	The TSC is the executive body for the trial. However, the TSC should have good reason before deciding not to accept requests from the TMG and recommendations from the DMC. If there are serious problems or concerns with the TSC decision following a DMC recommendation, a joint meeting of the TSC and DMC should be held. The information to be shown would depend upon the action proposed and each committee's concerns. Depending on the reason for the disagreement confidential data may have to be revealed to all or some of those attending such a meeting: this would be minimised where possible. The meeting would be Chaired by a senior member of the BTC or an external expert who is not directly involved with the trial.
Content	Details of TSC
10. After the trial	
Publication of results	The TSC will oversee the timely analysis, writing up and publication of the main trial results. The independent members of the TSC will have the opportunity to read and comment on the proposed main publications of trial data prior to submission. This review may be concurrent to that of the trial investigators and DMC.
The information about the TSC that will be included in published trial reports	TSC members will be named, and their affiliations listed in the main report, unless they explicitly request otherwise.
Any constraints on TSC members divulging information about their deliberations after the trial has been published	Unless permission is agreed with the DMC, the TSC will not discuss confidential information to what they have been privy to as a result of their involvement in the trial until 12 months after the primary trial results have been published. They should not trade in stock of companies affected by the trial until the results are public knowledge.

Figure 1: Relationships of Trial Committees

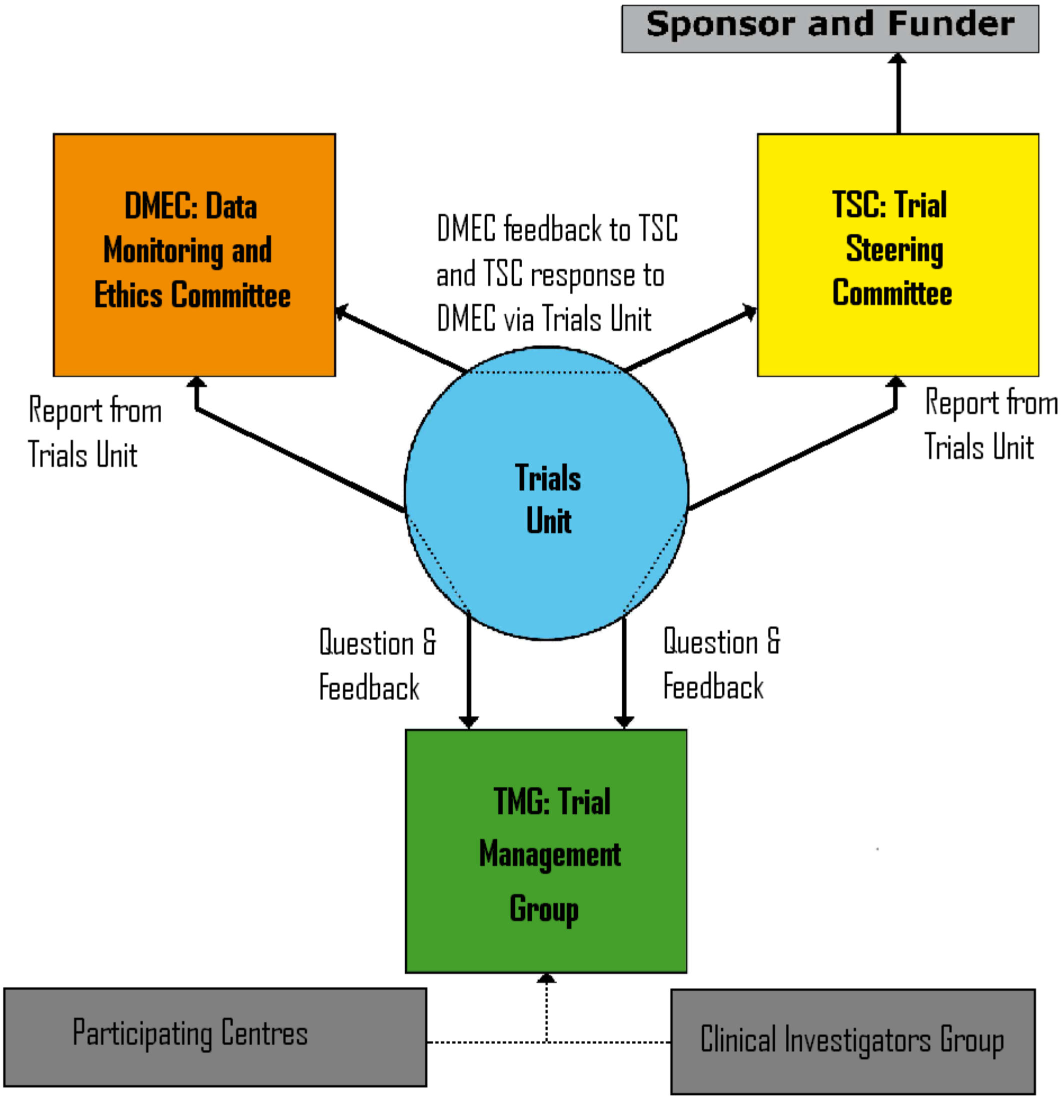

Please note that “Trials Unit” can be replaced by “Trial Manager” where appropriate.

Annexe 1: Agreement and competing interests form for independent members

TIGER Study (NIHR133464): Agreement to join the Trial Steering Committee as an independent member and disclosure of potential competing interests

Please complete the following document and return to the TSC Facilitator.
(please initial box to agree)

☐	I have read and understood the TSC Terms of Reference, Version 1.0, 23 September 2022
☐	I agree to join the Trial Steering Committee for this trial as an independent member
☐	I agree to treat all sensitive trial data and discussions confidentially

The avoidance of any perception that independent members of a TSC may be biased in some fashion is important for the credibility of the decisions made by the TSC and for the integrity of the trial.

Potential competing interests should be disclosed via the BTC. In many cases simple disclosure up front should be sufficient. Otherwise, the (potential) independent TSC member should remove the conflict or stop participating in the TSC. **Table 1** lists potential competing interests.

☐	**No**, I have no competing interests to declare
☐	**Yes**, I have competing interests to declare (please detail below)

Please provide details of any competing interests:

Name: ____

Signed:____Date:_________________________

Table 1: Potential competing interests for independent members

Consulting arrangements with the Sponsor/Funder
Frequent speaking engagements on behalf of the intervention
Career tied up in a product or technique assessed by trial
Hands-on participation in the trial
Involvement in the running of the trial
Emotional involvement in the trial
Intellectual conflict e.g. strong prior belief in the trial’s experimental arm
Involvement in the writing up of the main trial results in the form of authorship

Annexe 2: Agreement and competing interests form for non-independent members

TIGER Study (NIHR133464): Agreement to join the Trial Steering Committee as a non-independent member and disclosure of potential competing interests

Please complete the following document and return to the TSC Facilitator.
(please initial box to agree)

☐	I have read and understood the TSC Terms of Reference, Version 1.0, 23 September 2022
☐	I agree to join the Trial Steering Committee for this trial as a non-independent member
☐	I agree to treat all sensitive trial data and discussions confidentially

The avoidance of any perception that independent members of a TSC may be biased in some fashion is important for the credibility of the decisions made by the TSC and for the integrity of the trial.

Potential competing interests should be disclosed via the BTC. In many cases simple disclosure up front should be sufficient. Otherwise, the (potential) TSC member should remove the conflict or stop participating in the TSC. Table 1 lists potential competing interests.

☐	**No**, I have no competing interests to declare other than involvement in the trial
☐	**Yes**, I have competing interests to declare (please detail below)

Please provide details of any competing interests:

Name: ____

Signed:____Date:_________________________

Table 2: Potential competing interests for non-independent members

Consulting arrangements with the Sponsor/Funder<br>Frequent speaking engagements on behalf of the intervention<br>Intellectual conflict e.g. strong prior belief in the trial's experimental arm

Annexe 3: Agreement and confidentiality agreement for observers

TIGER Study (NIHR133464): Agreement to attend the Trial Steering Committee and treat all information confidentially

Please complete the following document and return to the Trial Manager.
(please initial box to agree)

☐	I agree to attend the Trial Steering Committee meeting on ___/____/______
☐	I agree to treat as confidential any sensitive information gained during this meeting unless explicitly permitted.

Name: ____

Signed: ____ Date:__________________________
